# Supplementary material for: Body image perception and physical activity behavior among adult population: Application of trans-theoretical model of behavior change
Source: PLoS One. 2024 Feb 26;19(2):e0297778. doi: 10.1371/journal.pone.0297778 (PMC10896515; doi:10.1371/journal.pone.0297778)
Supplement: S1 Table — (DOCX) [file pone.0297778.s001.docx]

**S1 Table: The full version of Marcus-TTM based on the stages of physical activity change**

| **Moderate Physical Activities** are defined as activities ranging between 3 - 6 METS. These activities require more oxygen consumption that light activities. Some examples of moderate physical activities include: sweeping the floor, walking briskly, slow dancing, vacuuming, washing windows, shooting a basketball.  **Vigorous Intensity Activities** are defined as activities ≥ 6 METS. Vigorous activities require the highest amount of oxygen consumption to complete the activity. Examples of vigorous physical activities include running, swimming, shoveling, soccer, jumping rope, carrying heavy loads (i.e. bricks). |
| --- |
| **According to the above definitions, you are currently in which of the following 5 situations?** *(just tick one of the options below)* |
| 1. I currently do not engage in moderate or vigorous PA on a regular basis, and I do not intend to start PA in the next 6 months (**Pre-contemplation**), Yes or No |
| 1. I am not currently engage in moderate or vigorous PA on a regular basis, but I plan to start PA in the next 6 months (**Contemplation**), Yes or No |
| 1. I am not currently engage in moderate or vigorous PA on a regular basis, but I plan to start in the next 6 months (**Preparation**), Yes or No |
| 1. I currently do moderate or vigorous PA on a regular basis, and I started it less than 6 months (**Action**), Yes or No |
| 1. I currently do moderate or vigorous PA on regular basis, and I started it more than 6 months (**Maintenance**), Yes or No |
