# Supplementary material for: Body image perception and physical activity behavior among adult population: Application of trans-theoretical model of behavior change
Source: PLoS One. 2024 Feb 26;19(2):e0297778. doi: 10.1371/journal.pone.0297778 (PMC10896515; doi:10.1371/journal.pone.0297778)
Supplement: S2 Table — (DOCX) [file pone.0297778.s002.docx]

**S2 Table: subject general characteristics**

| **Variables** | | **Total population**  **N=160** | **Total population**  **%** |
| --- | --- | --- | --- |
| **Age, *years***  ***M ± SD*** | Range: 22-55 | 36.21±8.25 |  |
| **Education** | Diploma | 56 | 32.9 |
|  | Bachelor | 64 | 37.6 |
|  | Master/PHD | 50 | 29.4 |
| **Education** | Diploma | 87 | 51.1 |
|  | Bachelor | 52 | 30.6 |
|  | Master/ PhD | 30 | 17.7 |
| **Marriage statues** | Single | 38 | 22.4 |
|  | Married | 132 | 77.6 |
| **Number of child** | 0 | 28 | 17.4 |
|  | 1 | 46 | 28.3 |
|  | 2 | 52 | 32.6 |
|  | 3 | 24 | 15.2 |
|  | More then 3 | 10 | 6.5 |
| **BMI** | Underweight | 2 | 1.2 |
|  | Normal | 86 | 50.6 |
|  | Overweight | 70 | 41.2 |
|  | Obese | 12 | 7.1 |
| **Stages of TTM** | Pre-contemplation | 25 | 20.7 |
|  | Contemplation | 48 | 28.6 |
|  | Preparation | 42 | 24.8 |
|  | Action | 23 | 13.35 |
|  | Maintenance | 23 | 13.36 |
| **BSRQ**  *M ± SD* | Appearance evaluation | 28.08 ± 3.9 |  |
|  | Appearance orientation | 52.11 ± 4.5 |  |
|  | Fitness evaluation | 11.35 ± 2.21 |  |
|  | Fitness orientation | 47.97 ± 7.16 |  |
|  | Health evaluation | 21.55 ± 3.15 |  |
|  | Health orientation | 32.14 ± 4.49 |  |
|  | Illness orientation | 20.74 ± 3.54 |  |
| **BASS**  *M ± SD* | Body area satisfaction | 36.28 ± 5.65 |  |
| **Attitude**  *^M ± SD^* | Self-classified weight | 6.02 ± 1.18 |  |
|  | Overweight reoccupation | 11.6 ± 1.4 |  |
| **Physical activity**  *M ± SD* | General score | 17.04±2.4 |  |
| **MBSRQ**  M ± SD | General score | 256.43 ± 23.45 |  |

±: Showing mean score (standard deviation); n: number of eligible participants; TTM: Trans-Theoretical Model; MBSRQ: Multidimensional Body-Self Relations Questionnaire.
