# Supplementary material for: Body image perception and physical activity behavior among adult population: Application of trans-theoretical model of behavior change
Source: PLoS One. 2024 Feb 26;19(2):e0297778. doi: 10.1371/journal.pone.0297778 (PMC10896515; doi:10.1371/journal.pone.0297778)
Supplement: S3 Table — (DOC) [file pone.0297778.s003.doc]

**S3 Table: Spearman Correlations Between MBSRQ subscale, PA, BMI, and demographic characteristics**

| Variables  (*n = 170*) | Age | Education | BMI | BASS | PA | BSRQ | Attitude | Number of child | MBSRQ |
| --- | --- | --- | --- | --- | --- | --- | --- | --- | --- |
| Age | 1 |  |  |  |  |  |  |  |  |
| Education | 0.155^*^ | 1 |  |  |  |  |  |  |  |
| BMI | 0.238^**^ | 0.014 | 1 |  |  |  |  |  |  |
| BASS | 0.012 | 0.108 | 0.233^**^ | 1 |  |  |  |  |  |
| PA | 0.079 | 0.043 | 0.016 | 0.290^**^ | 1 |  |  |  |  |
| BSRQ | 0.010 | 0.014 | 0.120 | 0.471^**^ | 0.605^**^ | 1 |  |  |  |
| Attitude | 0.131 | 0.179^*^ | 0.489^**^ | 0.132 | 0.043 | 0.078 | 1 |  |  |
| Number of child | 0.614^**^ | 0.016 | 0.174^*^ | 0.084 | 0.004 | 0.015 | 0.054 | 1 |  |
| MBSRQ | 0.018 | 0.007 | 0.137 | 0.630^**^ | 0.583^**^ | 0.978^**^ | 0.071 | 0.023 | 1 |

| ** Correlation is significant at the 0.01 level (2tailed).; * Correlation is significant at the 0.05 level (2tailed). |
| --- |
