# Supplementary material for: Body image perception and physical activity behavior among adult population: Application of trans-theoretical model of behavior change
Source: PLoS One. 2024 Feb 26;19(2):e0297778. doi: 10.1371/journal.pone.0297778 (PMC10896515; doi:10.1371/journal.pone.0297778)
Supplement: Attachment — Submitted filename: Response to reviewers.docx [file pone.0297778.s004.docx]

1. **Journal Requirements:**

| **When submitting your revision, we need you to address these additional requirements.** | |
| --- | --- |
| 1. Please ensure that your manuscript meets PLOS ONE's style requirements, including those for file naming. The PLOS ONE style templates can be found | Done |
| 2. In your Data Availability statement, you have not specified where the minimal data set underlying the results described in your manuscript can be found. PLOS defines a study's minimal data set as the underlying data used to reach the conclusions drawn in the manuscript and any additional data required to replicate the reported study findings in their entirety. All PLOS journals require that the minimal data set be made fully available. For more information about our data policy, please see <http://journals.plos.org/plosone/s/data-availability>. Upon re-submitting your revised manuscript, please upload your study’s minimal underlying data set as either Supporting Information files or to a stable, public repository and include the relevant URLs, DOIs, or accession numbers within your revised cover letter. For a list of acceptable repositories, please see http://journals.plos.org/plosone/s/data-availability#loc-recommended-repositories. Any potentially identifying patient information must be fully anonymized. Important: If there are ethical or legal restrictions to sharing your data publicly, please explain these restrictions in detail. Please see our guidelines for more information on what we consider unacceptable restrictions to publicly sharing data: http://journals.plos.org/plosone/s/data-availability#loc-unacceptable-data-access-restrictions. Note that it is not acceptable for the authors to be the sole named individuals responsible for ensuring data access. | All relevant data are within the paper and its Supporting Information files. The data was reported in this study is a portion of the large dataset of Iran Health care centres. The minimal data set necessary may only be used under the conditions laid down in the privacy regulations of the Iran Health Care centres. Therefore, the dataset is available on request. Data requests will be assessed against these regulations. Additionally, the minimal data set is available upon request from prof. Mohamad Vahedian (VahedianM@mums.ac.ir), project leader. |
| 3. PLOS requires an ORCID iD for the corresponding author in Editorial Manager on papers submitted after December 6th, 2016. Please ensure that you have an ORCID iD and that it is validated in Editorial Manager. To do this, go to ‘Update my Information’ (in the upper left-hand corner of the main menu), and click on the Fetch/Validate link next to the ORCID field. This will take you to the ORCID site and allow you to create a new iD or authenticate a pre-existing iD in Editorial Manager. Please see the following video for instructions on linking an ORCID iD to your Editorial Manager account | Done |
| 4. Your ethics statement should only appear in the Methods section of your manuscript. If your ethics statement is written in any section besides the Methods, please move it to the Methods section and delete it from any other section. Please ensure that your ethics statement is included in your manuscript, as the ethics statement entered into the online submission form will not be published alongside your manuscript. | It was added in the method section |
| 5. We note that Figures S1 and S3 in your submission contain map images which may be copyrighted. All PLOS content is published under the Creative Commons Attribution License (CC BY 4.0), which means that the manuscript, images, and Supporting Information files will be freely available online, and any third party is permitted to access, download, copy, distribute, and use these materials in any way, even commercially, with proper attribution. For these reasons, we cannot publish previously copyrighted maps or satellite images created using proprietary data, such as Google software (Google Maps, Street View, and Earth). For more information, see ourcopyright guidelines: We require you to either (1) present written permission from the copyright holder to publish these figures specifically under the CC BY 4.0 license, or (2) remove the figures from your submission: a. You may seek permission from the original copyright holder of Figures S1 and S2 to publish the content specifically under the CC BY 4.0 license. We recommend that you contact the original copyright holder with the Content Permission Form (http://journals.plos.org/plosone/s/file?id=7c09/content-permission-form.pdf) and the following text:“I request permission for the open-access journal PLOS ONE to publish XXX under the Creative Commons Attribution License (CCAL) CC BY 4.0 (http://creativecommons.org/licenses/by/4.0/). Please be aware that this license allows unrestricted use and distribution, even commercially, by third parties. Please reply and provide explicit written permission to publish XXX under a CC BY license and complete the attached form.”Please upload the completed Content Permission Form or other proof of granted permissions as an "Other" file with your submission.In the figure caption of the copyrighted figure, please include the following text: “Reprinted from [ref] under a CC BY license, with permission from [name of publisher], original copyright [original copyright year].”b. If you are unable to obtain permission from the original copyright holder to publish these figures under the CC BY 4.0 license or if the copyright holder’s requirements are incompatible with the CC BY 4.0 license, please either i) remove the figure or ii) supply a replacement figure that complies with the CC BY 4.0 license. Please check copyright information on all replacement figures and update the figure caption with source information. If applicable, please specify in the figure caption text when a figure is similar but not identical to the original image and is therefore for illustrative purposes only.The following resources for replacing copyrighted map figures may be helpful:USGS National Map Viewer (public domain): http://viewer.nationalmap.gov/viewer/The Gateway to Astronaut Photography of Earth (public domain): http://eol.jsc.nasa.gov/sseop/clickmap/Maps at the CIA (public domain): https://www.cia.gov/library/publications/the-world-factbook/index.html and https://www.cia.gov/library/publications/cia-maps-publications/index.htmlNASA Earth Observatory (public domain): http://earthobservatory.nasa.gov/Landsat: http://landsat.visibleearth.nasa.gov/USGS EROS (Earth Resources Observatory and Science (EROS) Center) (public domain): http://eros.usgs.gov/#Natural Earth (public domain): http://www.naturalearthdata.com/ | Based on the reviewer comment these figures was removed from this manuscript. |
| 6. Please upload a copy of Supporting Information Table S1 which you refer to in your text on page 13. | DONE |

2. **Additional Editor Comments:**

| We would like to thank you for the thoughtful comments and constructive suggestions, which help to improve the quality of this manuscript. We tried to answer to all valuable comments/suggestions/queries and all answers to comments are written within the document **by using Italic format**. This manuscript was edited to removed grammatical errors, and the edited part was written by G**reen colour.** | |
| --- | --- |
| **1. What are the inclusion criteria described on page 9? The population sample was all employees working in Binaloud's health care organizations (how many employees)?** | *The target population included employees working in health care if they met all the following inclusion criteria: (a) were adult population (over 18 years of age); (b) able to give informed consent; (c) did not suffer chronic disease or hospitalized in the past three months; and (d) did not have psychological disorders and sexual orientations. Participants were also excluded if they were pregnant and had not filled out all questionnaire items. To monitor people's eligibility, their medical records were first checked via Electronic Health Records (EHRs). These private and secure lifetime records explain an individual’s health history and care. This record system is made up of information from different sources (e.g., doctors, health care clinics, hospitals, laboratories, and pharmacies) that facilitate access to individual medical records, promote the accuracy of treatment decisions and the quality of care, improve clinical research, and reduce medical cost. After that, we interviewed the people and during the interview, we asked them verbally about their medical and mental status, and their weight was also calculated.*  *Since the target population includes all employees working in Binaloud's health care organizations, if meet inclusion criteria. A total of 216 employees worked in Binaloud's healthcare organizations, of which 192 met the inclusion criteria, and 22 did not answer all the items in the questionnaire. Finally, 170 employees (55 men and 115 women) were included in the data analysis and completed all questionnaires.* |
| **What instrument was used to determine stage of change? Citation #34 is incomplete on the reference page, however, it does not provide a stages of change instrument for physical activity.** | *The scale of change stages included of one question with five change stages (Marcus et al., 1992). The full version of Marcus-TTM based on the stages of physical activity change were examined using a 5-item based on dichotomous scale (no/yes) related to regular physical activity behaviour and intentions. Then, we categorized participants into one of the five stages of physical activity behaviour change explained earlier. For example, participants select, “I currently do not engage in moderate or vigorous physical activity on a regular basis, and I do not intend to start physical activity in the next 6 months” if they were in the precontemplation stage (Table S1). The CVR, CVI, and overall Cronbach’s alpha of the questionnaire was 0.95,0.87, and 90, which was acceptable in this study.* |
| **The description of the TTM is incomplete at best. Behavior change, as measured by the stages of change, is only different between preparation and action. There is not physical activity in pre-comtemplation contemplation or preparation. In the action and maintenance stages, participants are engaging in regularly activity and the only difference between the stages is how long (6-months) participants have been engaging in physical activity. The authors need to describe that they only focused on one variable of the TTM (stages of change).** | *Table 3 illustrates how the stages of the TTM are related to BMI, PA, and body image subscales. In each stage, the proportion of participants differed based on the criteria defined for moderate or vigorous physical activity. Individuals with no intensity, frequency, and duration criteria staged the greatest number of precontemplation and contemplation (30%) and the fewest number in maintenance (10.6%) or action (12.9%). The response format of TTM showed small increases in physical activity from precontemplation to preparation, while the level of physical activity significantly increased as individuals moved from preparation to the action stage.*  *Consistent with predictions of the TTM, physical activity is only different between the preparation and action stages because individuals are not engaging in physical activity in pre- contemplation, contemplation, and preparation stages (inactive stages) compared with the action and maintenance stages. In this study, a significant effect was observed from preparation to action points, the stages at which participants are engaging in regular physical. This result is in line with the same study that reported participants engaging in regular activity in the action and maintenance stages, and the difference is only related to times of physical activity between the stages. Surprisingly, our finding showed small increases in physical activity from pre-contemplation to preparation, suggesting that transitions could be correlated with changes in physical activity even among inactive stages.* |
| **Stages of changes is a categorical variable (mutally exclusive as participants by definition may be in only one stage) so it should not be analyzed using spearman correlations. If the authors believe (and can support theoretically) that the stages are rank ordered than they should make the case. Marital status is the same either yes or no? is there a rank order? Based on the title of table 4, it is unclear what correlation is being reported. Are they all spearman rank order correlations?** | *It was corrected .* |

**3. Review Comments to the Author**

| **Reviewer 1** |  |
| --- | --- |
| Body Image Perception, Weight Concerns and Physical Activity among Adult Population: Application of Trans-Theoretical Model The study on body image, BMI, and PA investigated across five stages of TTM, and tested associations with BMI, PA, and socio-demographic characteristics towards body image concerns in Iranian men and women. Despite sound results found in figure and tables, the methodology, results, and conclusion were not written well and strong. I recommend the authors to revise the manuscript based on major comments. | We would like to thank you for careful and thorough reading of this manuscript and for the thoughtful comments and constructive suggestions, which help to improve the quality of this manuscript. We tried to answer to all valuable comments/suggestions/queries and all answers to comments are written within the document by using **Red color.** This manuscript was edited to removed grammatical errors, and the edited part was written by G**reen colour.** |
| **Abstract** Methods: How TTM model was implemented in this study? (add these to the methods in abstract and main file).  Results: which sub subclasses of MBSRQ showed a significant difference should be added to the results. -Conclusion doesn’t show the main findings of the study. | Based on this comment abstract revised as follow: **Background:** The human body changes during life, but research on how sociodemographic characteristics and physical activity (PA) related to all aspects of body image are still unclear and there is conflict in the relevant results. This study aims to examine the relationships between all subclasses of body images and objectively measured BMI, PA, and sociodemographic characteristics. **Methods:** The study was a population-based cross-sectional survey of 170 health employees (55 men and 115 women) in Binaloud, Iran. Participants completed a self-administered questionnaire, which consisted of a self-administered Multidimensional Body-Self Image Questionnaire (MBSRQ) and stages of change scales. The full version of Marcus-TTM based on the stages of physical activity changes was used based on 5 items related to regular physical activity behavior and intentions. **Results:** There were no significant differences between men and women in total body image score, body area satisfaction, and attitude. Disease orientation (p=0.02) and health orientation (p= 0.05) were the only significant differences between men and women. The number of children, level of education, BMI, and PA had a stronger influence on body image concerns and body dissatisfaction. PA and 7 of the 10 subclasses of MBSRQ (appearance evaluation, fitness evaluation, fitness orientation, health evaluation, health orientation, and illness orientation) showed a significant difference (P<0.05) between participants in the five TTM stages. **Conclusion:** The ideal body image and body satisfaction may differ by the number of children, level of education, gender, BMI, and PA. Evidence from this study supports that participants’ stages of change affect their physical activity and body image perception. This result suggests a need for conducting work/home place intervention to promote adults’ body image perception and body area satisfaction based on the usage-specific stage under consideration. |
| Background: this part is so long and unrelated to the study objectives. I couldn’t find any related literature review to show the gap of knowledge for the study. Concepts and structure-based study related to TTM, physical activity, should be clearly added to the background. This part was not satisfying and written in weak pattern | Based on this comment most part of background re-written and under-relevant part were deleted. |
| Figures s 1 and s 2 is not related to this study and only shows the prevalence of outcomes…….. I recommended to remove both supplemental figures from the background. | It was removed |
| The number of questions used for TTM and samples should be added to the method. | The number of questions used for TMM and samples were added (Table s2). The scale of change stages included of one question with five change stages (Marcus et al., 1992). The full version of Marcus-TTM based on the stages of physical activity change were examined using a 5-item based on dichotomous scale (no/yes) related to regular physical activity behavior and intentions. Then, we categorized participants into one of the five stages of physical activity behaviour change explained earlier. For example, participants select, “I currently do not engage in moderate or vigorous physical activity on a regular basis, and I do not intend to start physical activity in the next 6 months” if they were in the precontemplation stage (Table S2). The CVR, CVI, and overall Cronbach’s alpha of the questionnaire was 0.95,0.87, and 90, which was acceptable in this study. |
| Method:in the first part of the method authors declared that all men (n= 92) and women (n= 124) working in Binaloud's health care. And in the next paragraph stated that “the target population includes all employees working in Binaloud's health care organizations, we used convenience sample to select eligible participants easy to contact and met inclusion criteria”. | It was corrected as follow: Since the target population includes all employees working in Binaloud's health care organizations, if meet inclusion criteria. A total of 216 employees worked in Binaloud's healthcare organizations, of which 192 met the inclusion criteria, and 22 did not answer all the items in the questionnaire. Finally, 170 employees (55 men and 115 women) were included in the data analysis and completed all questionnaires. |
| Results: Second line of the results after age, PA result need suitable unite of measurements. Please, add to the section as well as for the male. | We do not have a unit for physical activity in this study. The PA was examined using the five validated short questions on PA. It was rated on a 5-point Likert scale ranging from 1 (completely disagree) to 5 (completely agree). PA score was ranged from 5 to 25. |
| - Table 2, the name of TTM model with each stage should be written. | It was done. |
| How PA was measured? Which questions were used for the measurements? Please, explain it clearly. | The PA was examined using the five validated short questions that corresponds to the following questions: I do regular exercise, I am trying to increase my physical strength, it is important for me to participate in PA, I try to be physically active, and I tend to do regular exercise throughout the year. It was rated on a 5-point Likert scale ranging from 1 (completely disagree) to 5 (completely agree). The overall Cronbach’s alpha for these questions was 0.84. |

| **Reviewer 2** |  |
| --- | --- |
| The manuscript reports a study about the modification of body image and physical activity across ages in Iranian population. The paper presents specific interesting aspects like the evaluation of body image attitudes in understudied population. The results showed that body image issues are like Western countries. However, I think the authors should revised their paper looking at specific aspects that are missing in their paper: | We would like to thank you for careful and thorough reading of this manuscript and for the thoughtful comments and constructive suggestions, which help to improve the quality of this manuscript. We tried to answer to all valuable comments/suggestions/queries and all answers to comments are highlighted with **yellow colour**. |
| The introduction is quite long and not focused on the paper's goal. Please revised this aspect. | Based on this valuable comment most part of background re-written and under-relevant parts were deleted. |
| - have you evaluated psychological and medical conditions? because these aspects might have a role in body image issues. For example, eating disorders have specific effects on body image evaluation and perception, as well as bariatric surgery or extreme obesity, see <https://doi.org/10.1002/erv.2812> and <https://doi.org/10.1007/s11695-020-05166-z/> have you evaluated sexual orientations? Because this aspect has been shown to be crucial for body image concerns, especially in the male/female comparisons, see <https://doi.org/10.1007/s40519-020-01047-7>/is it possible that physical activity and body image concerns are linked in a compulsory way like it happens in eating disorders (see <https://doi.org/10.1016/j.eatbeh.2022.101675>)? The idea that people use physical activities to modulate their body image concerns is very closed to what has been shown in Western countries and it is very interesting that also your study showed that. | Although, a significant proportion of clinical studies indicated psychological and medical conditions, obesity, and sexual orientations may affect body image concern and perception, the literature review highlighted the need for further research to examine all aspects of objectively measured and self-reported body image and body dissatisfaction, in the context of the socio-demographic characteristics of different adult groups. Therefore, the main target in this study was applied the TTM to examine gender-specific body image perception and physical activity in relation to BMI and sociodemographic characteristics among health employees who worked in health and medical care centres. In this study, we assume that adult males and females with different sociodemographic characteristics have significantly different body image perception and PA behavior. So, psychological and medical conditions were not considered in this study. The target population included employees working in health care if they met all the following inclusion criteria: (a) were adult population (over 18 years of age); (b) able to give informed consent; (c) did not suffer chronic disease or hospitalized in the past three months; and (d) did not have psychological disorders and sexual orientations. Participants were also excluded if they were pregnant and had not filled out all questionnaire items. |
| have you evaluated gender or sex? please be consistent with this through the manuscript | Yes, the main target in this study was applied the TTM to examine gender-specific body image perception and physical activity in relation to BMI and sociodemographic characteristics among health employees who worked in health and medical care centres. So, all analysis were conducted based gender differences and also it was considered in discussion. |
| you reported only obesity as problem related to body, but recent studies have showed the presence also of high rates of eating disorders in the Middle East Countries. Has this aspect been considered? | It seems that the associations between PA and body image are dynamic, complex, and bidirectional[20] because various aspects might have specific effect on PA and body image concerns such as eating disorders, psychological and medical conditions, bariatric surgery, and sexual orientations. This issue could be considered as new research questions of focus for future study. |
|  |  |
